# Errors in Antimicrobial Prescription and Administration in Very Low Birth Weight Neonates at a Tertiary South African Hospital

**DOI:** 10.3389/fped.2022.838153

**Published:** 2022-03-03

**Authors:** Sandi L. Holgate, Adrie Bekker, Veshni Pillay-Fuentes Lorente, Angela Dramowski

**Affiliations:** ^1^Department of Paediatrics and Child Health, Faculty of Medicine and Health Sciences, Stellenbosch University, Cape Town, South Africa; ^2^Division of Clinical Pharmacology, Department of Medicine, Faculty of Medicine and Health Sciences, Stellenbosch University, Cape Town, South Africa

**Keywords:** antimicrobial, prescription error, very low birth weight, neonatal sepsis, adverse event

## Abstract

**Background:**

Antimicrobial prescription and administration-related errors occur frequently in very low birth weight (VLBW; <1,500 g) neonates treated for bloodstream infections (BSI).

**Methods:**

Antimicrobial prescriptions for the treatment of laboratory-confirmed BSI were retrospectively analyzed for VLBW neonates at Tygerberg Hospital, Cape Town, South Africa (1 July 2018 - 31 December 2019), describing antimicrobial type, indication, duration of therapy and BSI outcomes. The prevalence of, and risk factors for prescription (dose, interval) and administration errors (hang-time, delayed/missed doses) were determined.

**Results:**

One hundred and sixty-one BSI episodes [16 (9.9%)] early-onset, 145 [90.1%] healthcare-associated) affected 141 neonates (55% male, 25% born to mothers living with HIV, 46% <1,000 g birth weight) with 525 antimicrobial prescription episodes [median 3.0 (IQR 2–4) prescriptions/BSI episode]. The median duration of therapy for primary BSI, BSI-associated with meningitis and BSI-associated with surgical infections was 9, 22, and 28 days, respectively. The prevalence of dose and dosing interval errors was 15.6% (77/495) and 16.4% (81/495), respectively with prescription errors occurring most commonly for piperacillin-tazobactam and vancomycin given empirically. Administration errors were less frequent [3.8% (219/5,770) doses missed; 1.4% (78/5,770) delayed], however 64% had a hang-time (time from sepsis diagnosis to 1st dose of antimicrobial) exceeding 60 min. On multivariable analysis, postnatal age >7 days was associated with prescription errors (*p* = 0.028). The majority of neonates with BSI required escalation of respiratory support (52%) and 26% required intensive care admission. Despite fair concordance between empiric antimicrobial/s prescription and pathogen susceptibility (74.5%), BSI-attributable mortality in this cohort was 30.4%.

**Conclusion:**

VLBW neonates with BSI's were critically ill and had high mortality rates. Hang-time to first antimicrobial administration was delayed in two-thirds of BSI episodes and prescription errors affected almost 1 in 6 prescriptions. Targets for intervention should include reducing hang-time, use of standardized antimicrobial dosing guidelines and implementation of antimicrobial stewardship recommendations.

## Introduction

In Sub-Saharan Africa (SSA), ~1 million deaths occur in the neonatal period annually (1), with prematurity, intrapartum related events and infection as the leading causes (2). The case fatality rate of 14% for bloodstream infections (BSI) (3) may be an underestimate given recent data suggesting that many neonatal deaths currently attributed to prematurity are actually caused by undetected infection, often with antimicrobial-resistant bacteria (4).

In SSA, gram negative organisms, with increasing antimicrobial resistance rates, predominate as causative pathogens for neonatal sepsis and meningitis (5–9). Due to limited availability of antimicrobials for resistant neonatal infections (10), it is of utmost importance to ensure antimicrobials are correctly used in this vulnerable population.

Evidence from sepsis trials in adult, pediatric and neonatal populations gave rise to the concept of the “golden hour”, recommending receipt of the first dose of empiric antibiotic/s within 1 h of presentation to improve sepsis outcomes (11, 12).

Physiology in the neonatal period is constantly changing and developing requiring frequent dose and interval changes to achieve adequate drug concentrations. These physiological changes may affect the pharmacokinetics and pharmacodynamics of drugs resulting in the need for dosing adjustments in accordance with age, weight and/or body surface area (13–15), increasing the potential for prescribing errors.

Neonates, particularly those in the intensive care setting, are at high risk of medication errors (16–18), many due to poor prescribing (19). Antimicrobials are among the most common drug classes to be associated with medication errors (20–22), with up to a third resulting in harm to the patient (21). Dosing errors, inappropriate antimicrobial for the underlying condition, premature cessation, laboratory monitoring errors and therapeutic duplication are frequent errors, with vancomycin and piperacillin-tazobactam being the agents most commonly associated with prescription errors (23). Some antimicrobials (e.g., aminoglycosides and vancomycin) have toxic side effects and a narrow therapeutic index (24), necessitating therapeutic drug monitoring (TDM) to protect patients from these harms, and guide optimal and adequate antimicrobial dosing (25).

Given the limited data from SSA regarding this widespread problem we reviewed the prevalence of, and contributors to, antimicrobial prescription and administration errors in very low birth weight (VLBW: <1,500 g) neonates with laboratory-confirmed BSI episodes at a large South African neonatal unit.

## Methods

### Study Design and Population

We conducted a retrospective, descriptive study of antimicrobial prescription- and administration-related errors in VLBW neonates with laboratory-confirmed bacterial and fungal BSI at Tygerberg Hospital, Cape Town, South Africa between 1 July 2018 and 31 December 2019. Inborn neonates with a birthweight of 500–1,500 g who developed culture-confirmed bloodstream infection during their hospital stay were eligible for inclusion. This weight band was selected due to the higher number of BSI's seen in this group (26) and complexity in prescribing for smaller neonates. BSI was diagnosed by incubating at least 1 ml of aseptically collected blood into BacT/Alert PF Plus bottles (Biomérieux, Marcy l'Ètoile, France). Study exclusion criteria were: patients transferred in from outlying facilities as antimicrobials may have been administered before transfer; neonates with growth of pathogens on urine, tracheal aspirate or wound swab cultures only; neonates with blood and cerebrospinal fluid (CSF) cultures isolating contaminants as defined by the United States Centre for Disease Control and Prevention list of commensal flora (27), and neonates receiving antiviral medications.

### Study Setting

Tygerberg Hospital is the tertiary referral hospital for sick and/or preterm neonates requiring specialist medical and/or surgical care in Cape Town's Metro East. The neonatal unit comprises four 30-bed wards and a 12-bed medical and surgical neonatal intensive care unit (NICU), with a 132-bed total capacity and occupancy rates averaging 93%. Owing to a shortage of NICU beds in the Western Cape Province, a provincial periviability policy was developed to guide decisions on eligibility for NICU admission (generally >800 g and >27 weeks gestation) (28). Standard practice in this setting is for all sick and/or preterm neonates to be admitted into a high care ward where respiratory support such as nasal continuous airways pressure (nCPAP) or high flow nasal cannula can be initiated, surfactant administered, intercostal drains inserted and central lines placed for total parenteral nutrition (TPN) if needed. NICU is reserved for neonates requiring mechanical ventilation, therapeutic hypothermia or inotropic support.

### Empiric Antimicrobial Therapy for Neonatal Infections

For early onset (within 72 h of birth) sepsis, ampicillin and gentamicin are the empiric antimicrobial of choice. In neonates older than 72 h of life where healthcare-associated infection (HAI) is suspected (26), piperacillin-tazobactam plus amikacin is recommended, unless the baby is clinically unstable or there is a clinical suspicion of a gram negative infection or meningitis. In these cases, meropenem is recommended to provide cover for antimicrobial resistant pathogens and to enhance CSF penetration. Vancomycin is added if the neonate has or had a central venous catheter inserted or if a skin or soft tissue infection is present, where methicillin-resistant *S. aureus* is a likely pathogen. Antifungal therapy is added where clinically indicated and colistin is used in cases where a carbapenem-resistant organism is very likely (empiric therapy) and/or microbiologically-confirmed (targeted therapy). Antimicrobial prescriptions are written by all levels of medical doctors, from juniors (medical officers and residents) to seniors (fellows, consultant Pediatricians and neonatologists). A specific antimicrobial prescription chart is used which allows for the drug name, dose, route of administration, frequency, start and stop date and the prescribing doctors name and signature. Registered nurses are responsible for preparing and administering the antimicrobials. All antimicrobial therapy for bloodstream infection is administered intravenously in this population.

### Therapeutic Drug Monitoring for Antimicrobial Agents

Locally, trough TDM is recommended before the 3rd dose of gentamicin and amikacin to limit potential toxicities (29). In mid-2019 local vancomycin dosing guidelines were changed to recommend a loading dose of vancomycin followed by maintenance doses which vary according to gestational age in preterm infants (29). Prior to this change TDM was recommended before the 4th dose, whereas later TDM samples were recommended before administration of the 3rd or 4th vancomycin dose. When reviewing the prescriptions, decision regarding correct timing of the TDM was based on whether the loading dose was given. The Division of Clinical Pharmacology uses DoseMeRx software (30) to predict vancomycin dose based on gestational age, weight, change in creatinine concentration and a vancomycin trough concentration.

### Study Objectives and Definitions

Our primary objective was to determine the prevalence of antimicrobial-related medication errors in VLBW neonates with laboratory-confirmed BSI. Duplicate BSI pathogens isolated within 10 days of the first positive culture, were considered to represent a single infection episode. Coagulase-negative staphylococci (CoNS) were considered to be pathogens, if 2 or more CoNS were isolated from blood cultures drawn within two consecutive days. Data were analyzed comparing neonates with early onset sepsis (EOS) to those with healthcare-associated BSI (HA-BSI episodes that developed >72 h after admission to hospital).

For clinical impact of the BSI event we described: escalation of respiratory support and/or need for mechanical ventilation, admission to NICU, inotropic support (24 h prior to and up to 72 h after sepsis diagnosis) and need for blood products or surgery during antimicrobial therapy. In a study population such as VLBW neonates, where co-morbitidies may be high, BSI attributable death [death within 72 h of BSI (31)] was compared to those who died more than 72 h after the blood culture (BSI associated death) in attempt to clarify the contribution of the BSI to the mortality rate (32).

The indication for antmicrobials and the overall antimicrobial treatment duration was determined. We calculated the frequency of dose errors (a 10% cut off margin on either side of the ideal dose was used) (33), dosing interval errors and missed or delayed (>1 h) antimicrobial doses. Two local reference books, compiled from international literature by the two tertiary hosipatls in the Western Cape were used (29, 34). We also determined the hang-time (interval from clinical infection diagnosis, based on the time of blood culture sampling, to administration of the first dose of antimicrobial) to empiric and concordant antimicrobial therapy. One hour was used as a target hang-time based on the increased mortality associated with every hour delay in initiating appropriate antimicrobials (11). Cases where death occurred before antimicrobial administration and where the first dose of antimicrobial was documented as given before infection diagnosis were excluded from the hang-time analysis. The proportion of discordant empiric antimicrobial prescriptions was described i.e., mismatch between the pathogen identified and its sensitivity profiles to the empiric antimicrobial prescribed. We described the proportion of antimicrobial prescription episodes where TDM sampling was not performed at the recommended time, according to the local guidelines. For this we reviewed notations on the prescription chart in combination with data from the pharmacology laboratory. We assessed the proportion of TDM values in the therapeutic drug concentration range and proportion of TDM episodes where dose adjustments were enacted (if required). In cases where there was more than 1 prescription for vancomycin per BSI, repeat prescriptions were not analyzed for prescription errors as dose or interval changes may have been made according to TDM recommendations.

### Data Sources

Data were collated from the Tygerberg Hospital Vermont Oxford Network neonatal database, the hospital's electronic record management system, the National Health Laboratory Service (NHLS), Tygerberg Hospital Clinical Pharmacology Laboratory and Unit for Infection Prevention and Control's neonatal BSI surveillance records. Patient records were reviewed to collect demographic, clinical, laboratory and antimicrobial prescription data. Data were entered into an institutional-hosted REDCap (Research Electronic Data Capture) database (35).

### Statistical Analysis

The sample size required to deliver a 95% confidence interval with a + or – 5% margin of error at an estimated prescribing/administration error prevalence of 20% was calculated at 111 patients with infection episodes. Descriptive analyses of neonatal and maternal demographic characteristics were performed. Continuous variables (e.g., days of antibiotic therapy) were reported as median with interquartile ranges (IQR), and categorical data (e.g., missed antibiotic doses) were reported as proportions/percentages. Students *t*-test, Pearson's chi squared test and other non-parametric tests (e.g., Fishers Exact) were used for hypothesis testing where appropriate. Demographic factors associated with prescription errors were evaluated using a multivariate binomial regression model where significant univariate predictors were added at a *p*-value of 0.2 to inform the multivariate model. For all statistical tests performed, a *p*-value < 0.05 was considered significant. All the statistical analyses were performed using STATA 16.0 (College Station, Texas 77845, USA).

Ethics approval for this study was received from the Stellenbosch University Health Research Ethics Committee, reference number N19/12/159 and Tygerberg Hospital via the National Health Research Ethics Committee.

## Results

### Maternal and Neonatal Characteristics

**Figure 1:**
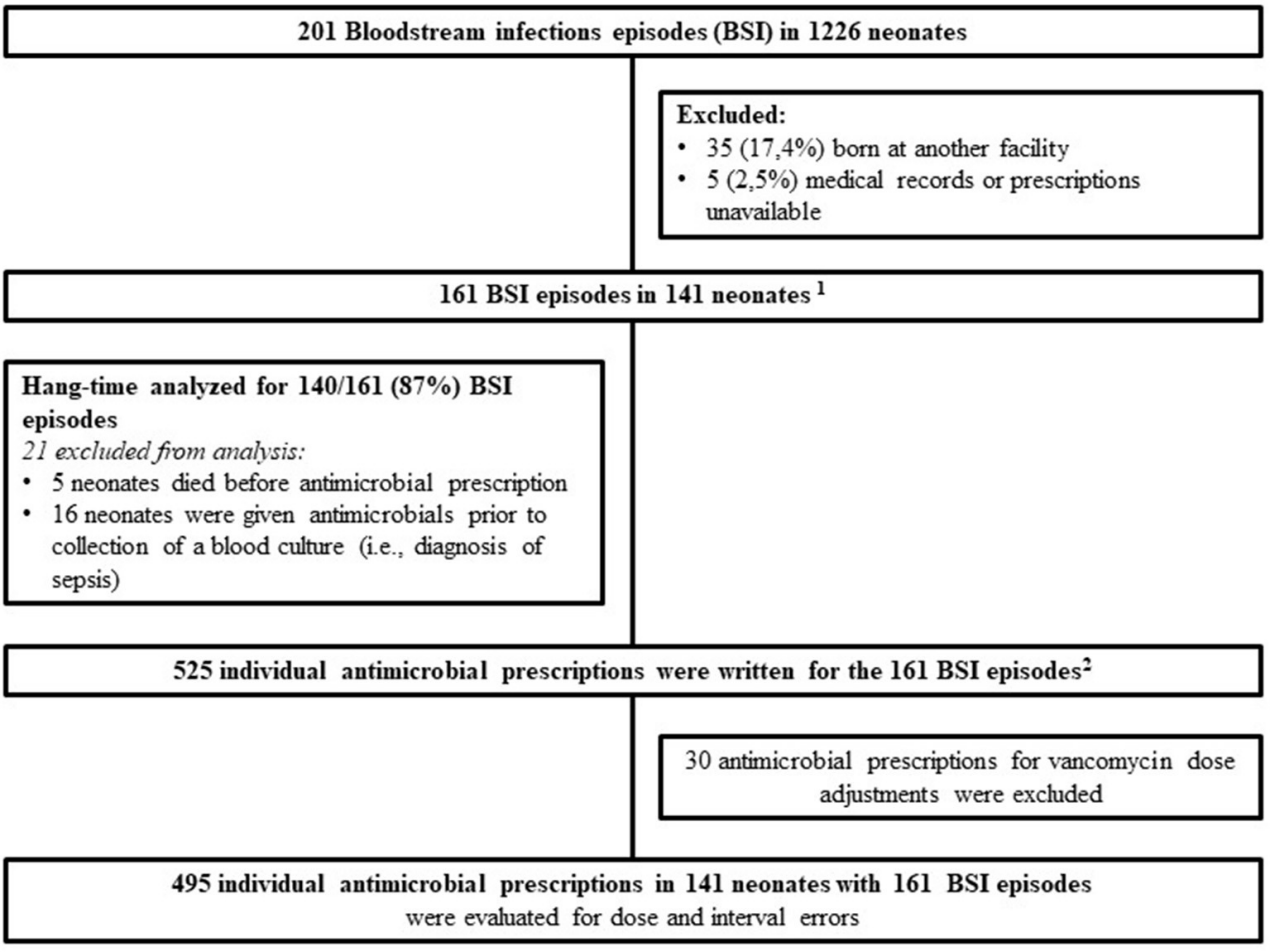
Culture confirmed blood stream infection episodes and prescriptions in very low birth weight neonates from 1 July 2018 to 31 December 2019. ^1^141 neonates: 15 with 2 BSI episodes, 1 with 3 BSI episodes and 1 with 4 BSI episodes. ^2^Missed and delayed doses were evaluated on all 525 prescriptions with the denominator being the total number of doses administered.

Two-hundred and one laboratory confirmed BSI episodes were identified in 1,226 VLBW neonates admitted to Tygerberg Hospital during the study period. Antimicrobial prescriptions were analyzed for 161 BSI episodes diagnosed in 141 neonates (Figure 1). The median gestational age and birth weight were 28 weeks and 1,030 g with a male predominance (78; 55.3%) (Table 1). A quarter of mothers were living with HIV. Forty four percent (62/141) went into spontaneous preterm labor and the remainder delivered following induction of labor or cesarean section for maternal indications. Of the 161 BSI episodes, the majority were healthcare associated (145; 90%) and occurred at a median of 10 (IQR 7–21) days of life (Table 2).

**Table 1:** Characteristics of mothers and very low birth weight neonates receiving antimicrobials for blood stream infection episodes (*n* = 141).

**Characteristic**	**Total neonates** ***N*** **= 141**
Sex, male, *n* (%)	78 (55.3)
Gestational age at birth in weeks, median (IQR)	28 (27–29)
Birth weight in grams, median (IQR)	1,030 (895–1,160)
**Birthweight categories**	
500–749 g, *n* (%)	10 (7.1)
750–999 g, *n* (%)	55 (39)
1,000–1,249 g, *n* (%)	54 (38.3)
1,250–1,500 g, *n* (%)	22 (15.6)
**Mode of delivery**	
Normal vertex delivery, *n* (%)	46 (32.6)
Cesarean section, *n* (%)	95 (67.4)
Mother attended antenatal care^a^, *n* (%)	132 (93.6)
**Maternal HIV status**	
HIV-negative, *n* (%)	103 (73.1)
Women living with HIV, on antiretroviral therapy (ART), *n* (%)	35 (24.8)
Women living with HIV, not on ART, *n* (%)	1 (0.7)
HIV status unknown, *n* (%)	2 (1.4)
**Maternal complications and treatment**	
Any antenatal steroids received, *n* (%)	127 (90.1)
Maternal chorioamnionitis, *n* (%)	2 (1.4)
Maternal urinary tract infection, *n* (%)	7 (5.0)
Spontaneous preterm labor, *n* (%)	62 (44.0)
Prolonged rupture of membranes (>18 h), *n* (%)	23 (16.3)

a *Maternal antenatal care was defined as documented attendance of any prenatal obstetric care prior to delivery. IQR, interquartile range*.

**Table 2:** Antimicrobial therapy and impact of blood stream infection episodes (161 episodes in 141 neonates)—a comparison between early onset and healthcare associated BSI.

**Antimicrobial therapy and impact**	**Total BSI episodes** ***N*** **= 161**	**Early-onset BSI episodes** ***N*** **= 16**	**Healthcare-associated BSI episodes** ***N*** **= 145**	***P*** **-value**
Age in days at infection onset, median (IQR)	10 (6–18)	0 (0–2)	10 (7–21)	N/A
**Indication for antimicrobials**, ***n*** **(%)**				
Primary BSI	94 (58.4)	11 (68.8)	83 (57.2)	N/A
BSI with proven meningitis	14 (7.7)	2 (12.5)	12 (8.3)	
BSI with necrotising enterocolitis	27 (16.8)	0 (0)	27 (18.6)	
BSI with catheter associated sepsis^a^	16 (9.9)	3 (18.8)	13 (9.0)	
BSI with other surgical source^b^	5 (3.1)	0 (0)	5 (3.4)	
More than 1 infection^c^	5 (3.1)	0 (0)	5 (3.4)	
**Duration of antimicrobial therapy course for infection episodes (days), median (IQR)^d^**				
Primary BSI	9 (5–13)	9 (5–10)	9 (6–14)	N/A
BSI with proven meningitis	22 (18–24)	19 (14–24)	22 (19.5–24.5)	
BSI with necrotising enterocolitis	2 (1–8)	–	2 (1–8)	
BSI with catheter associated sepsis^a^	10 (2–13)	1 (1–11)	10 (2–13.5)	
BSI with other surgical source^b^	28 (7–31)	–	28 (7–31)	
More than 1 infection^c^	22 (21–23)	–	22 (21–23)	
**Impact of BSI episode^e^**, ***n*** **(%)**				
Required escalation of respiratory support	84 (52.2)	5 (31.3)	79 (54.5)	N/A
Required NICU admission from HC ward	42(26.1)	5 (31.3)	37 (25.5)	
Required mechanical ventilation^f^	58 (36.0)	6 (37.5)	52 (35.9)	
Required inotropes	32 (19.9)	5 (31.3)	27 (18.6)	
Required blood products^g^	65 (40.4)	2 (12.5)	63 (43.4)	
Required surgery^g^	9 (5.6)	0 (0)	9 (6.2)	
**Outcome of BSI episodes**, ***n*** **(%)**				
Died within 72 h (BSI-attributable deaths)	49 (30.4)	6 (37.5)	43 (27.7)	0.57
**Antimicrobial prescriptions**				
Antimicrobial prescriptions for culture-confirmed BSI episodes, *n*	525	46	479	–
Antimicrobials prescribed per BSI episode, median (IQR)	3 (2–4)	3 (2–4)	3 (2–4)	–
**Appropriateness of the empiric prescription**, ***n*** **(%)**				
Concordant^h^	120 (74.5)	12 (75.0)	108 (74.5)	0.524
Discordant	36 (22.4)	2 (12.5)	34 (23.4)	
Died prior to receiving antimicrobial therapy	5 (3.1)	2 (12.5)	3 (2.1)	

*BSI, blood stream infection; SD, standard deviation; IQR, interquartile range; NICU, neonatal intensive care unit; HC, high care*.

a *Catheter associated sepsis = a neonate with clinical signs of sepsis plus a positive blood culture in the period between catheter insertion and 48 h post removal, with no other focus of sepsis*.

b *Five patients had non-NEC surgery e.g., joint aspiration for septic arthritis*.

c *More than 1 infection was when there was more than 1 source e.g., meningitis plus NEC*.

d *Those where patient demised before the 1st dose of antimicrobial could be administered were excluded*.

e *Escalation of care within 24 h before and 72 h after the positive blood culture*.

f *Includes those on mechanical ventilation at the time of sepsis diagnosis*.

g *Need for blood products or surgery at any time while the neonate was receiving antimicrobials*.

h *Concordance was when the organism cultured was sensitive to the empiric antibiotic prescribed*.

### Blood Stream Infection Episodes

Most BSI episodes were primary (no identifiable source) (94; 58.4%), with the remaining 67 (41.6%) comprising BSI with either meningitis, necrotising enterocolitis or other surgical source and catheter-associated BSI. Gram negative BSI pathogens predominated [117; 72.7%; 37% (59/161) Klebsiella species, 17% (26/161) *Serratia marcescens*], followed by gram positives [41; 25.5%; 16% (25/161) *Staphlococcus aureus*] and fungi (3; 1.9%). More than half (52%) of neonates needed escalation of respiratory support and 26% required admission to NICU. An additional 11% (18/161) were already admitted in the NICU at the time of the sepsis diagnosis. Thirty percent of BSI episodes (49/161) resulted in death within 72 h of the sepsis diagnosis, with a median (IQR) interval from blood culture collection to death of 1 (0–2) day.

### Prescription Episodes

A total of 525 individual antimicrobial prescriptions were identified for the 161 neonatal, including re-prescribing for dose and/or dosing interval changes (Table 2). A median of 3 (IQR 2–4) individual antimicrobial prescriptions were observed for each neonatal BSI episode. The median number of antimicrobial classes prescribed per episode was 3 (IQR 2–4), however fewer classes of antimicrobial were prescribed in the EOS group [median 2 (IQR 2–3) vs. 3 (IQR 2–4) in those with HA-BSI]. The median duration of antimicrobial therapy was shortest for primary BSI and longest for BSI associated with meningitis and surgical infections at 9, 22, and 28 days, respectively. In three-quarters of BSI episodes, the empiric antimicrobial therapy prescribed was concordant with the antimicrobial susceptibility of the BSI pathogen isolated (120; 74.5%). Discordance between pathogen and empiric antimicrobial did not significantly impact sepsis attributable mortality rates [9/44 (20%) vs. 27/112 (24%), *p* = 0.6, OR 0.8 (95% CI 0.3–1.9)] or 30 day mortality rates [11/58 (19%) vs. 25/98 (26%), *p* = 0.3. OR 0.7 (CI 0.3–1.5)].

### Hang-Time

Of the 161 BSI episodes included in the analysis, hang-time could only be determined for 140 (87%) BSI episodes, as 5 neonates died before AMO could be prescribed and in 16 neonates the AMO was administered prior to the diagnosis of sepsis. The median antimicrobial hang-time was 115 (IQR 48–210) min and did not differ by infection type (EOS vs. HA-BSI) (Table 3). Only 51/140 (36.4%) evaluable neonatal BSI episodes had administration of the first dose of empiric antimicrobials within 60 min. There was no difference in the proportion of neonatal BSI episodes with hang-time <60 min by BSI type [4/14 (28.6%) of EOS vs. 47/127 (37.0%) of HA-BSI; *p* = 0.770]. Furthermore, hang-time of <60 min was not associated with a reduced risk of sepsis attributable death [16/42 (38%) vs. 33/98 (36%); *p* = 0.789, OR 1.1 (95% CI 0.5–2.3)].

**Table 3:** Antimicrobial prescription errors and hang-time.

**Prescription errors and hang-time**	**Total prescription episodes** ***N*** **= 495**	**Prescription episodes for early-onset BSI** ***N*** **= 46**	**Prescription episodes for healthcare-associated BSI** ***N*** **= 449**	***P*** **- value**
Antimicrobials prescribed with incorrect dosage^a^, *n* (%)	77 (15.6)	0 (0%)	77 (17.2)	0.001
Antimicrobials prescribed with incorrect dosing interval^a^, *n* (%)	81 (16.4)	3 (6.5)	78 (17.4)	0.037
Hang-time in minutes to receipt of empiric antimicrobial/s, median (IQR)^b^	115 (48–210)	109.5 (13–120)	115 (50–240)	0.298
Hang-time in minutes to receipt of concordant antimicrobial, median (IQR)^b^	150 (60–658)	110 (53–140)	170 (60–790)	0.090

a *Where there was >1 prescription episode for vancomycin the subsequent prescriptions were excluded as adjustments may have been made according to the therapeutic drug monitoring*.

b *Patients who died before antimicrobials could be given and those where antimicrobial was recorded as given before the blood culture was taken were excluded from the hang time analysis*.

*IQR, interquartile range; BSI, bloodstream infections*.

### Prescription and Administration Errors

After excluding neonatal BSI epsiodes where changes to vancomycin prescriptions were needed based on TDM results (*n* = 30), 495 prescription episodes remained for analysis of dose and dose interval errors. Dose [0/46 (0%) EOS vs. 77/449 (17.2%) HA-BSI, *p* = 0.001] and dosing interval [3/46 (6.55) EOS vs. 78/449 (17.4%) HA-BSI, *p* = 0.037] prescription errors occurred significantly more frequently among neonates with HA-BSI than EOS (Table 3). Of the antimicrobials prescribed as empiric therapy for suspected BSI, vancomycin had the highest frequency of dosing errors (30.6%), whereas piperacillin-tazobactam had the highest frequency of dose interval errors (40.5%) (Table 4). Of the 495 prescriptions analyzed, 33 (6.7%) had inappropriately high doses and 44 (8.9%) had low doses. There were no incorrect doses in the prescriptions for EOS and only 3 dose interval errors in this group. Postnatal age > 7 days, compared to those older than 7 days, was the only significant factor associated with prescription errors [adjusted odds ratio 2.64 (95% CI 1.2–6.3); *p* = 0.028] (Table 5). Among all prescriptions, there were 219 (3.8%) missed doses and 78 (1.4%) delayed doses: 12 (3.5%) were delayed in the EOS group vs. 66 (1.2%) in the HA-BSI group; *p* = 0.001. We assessed gestational age, weight at the time of BSA, postnatal age in days and timing of infection as possible factors associated with admin errors but none were deemed significant. Compliance with adhernence to TDM recommendations was variable: 85% for vancomycin; 30% for amikacin and 13% for gentamicin (Table 6).

**Table 4:** Frequency of dosing and dosing interval errors for frequently used antimicrobials.

**Antimicrobial name^a^**	**Number of prescriptions**	**Frequency of dose errors, *n* (%)**	**Frequency of dose interval errors *n* (%)**
Ampicillin	30	0 (0)	6 (20.0)
Amikacin	78	18 (23.1)	3 (3.8)
Cefazolin	8	6 (75.0)	8 (100)
Cefotaxime	9	3 (33.3)	4 (44.4)
Colistin	21	7 (33.3)	3 (14.3)
Fluconazole	17	2 (11.8)	0 (0)
Gentamicin	14	1 (7.1)	0 (0)
Imipenem	5	1 (20.0)	3 (60.0)
Linezolid	5	1 (20.0)	1 (20.0)
Meropenem	136	5 (3.7)	1 (0.7)
Piperacillin-tazobactam	79	2 (2.5)	32 (40.5)
Trimethoprim/sulfamethoxazole	10	5 (55.6)	3 (33.3)
Vancomycin	72	22 (30.6)	15 (20.8)

a *Antimicrobials with fewer than five prescription episodes were excluded (amphotericin B, cephalexin, cefepime, ceftriaxone, cefuroxime, ciprofloxacin, clindamycin, ertapenem, penicillin G, rifampicin, tobramycin)*.

**Table 5:** Predictors of antimicrobial prescription errors in very low birth weight neonates.

**Variable**	**Unadjusted**	**Adjusted**
	**Odds ratio (95% CI)**	**Odds ratio (95% CI)**
**Gestational age**
≥28 weeks	0.7 (0.36–1.34)	–
**Highest weight at time of BSI**
≥1,000 g	0.8 (0.4–1.6)	–
**Postnatal age**
≥7 days	3 (1.4–6.3)	2.64 (1.17–6.29)
**Timing of infection**
Healthcare-associated BSI*	3.2 (0.9–10.7)	1.47 (0.36–5.95)

*BSI, bloodstream infections; CI, confidence interval*.

* *Healthcare-associated BSI was compared to early onset sepsis*.

**Table 6:** Therapeutic drug monitoring (TDM) of selected antimicrobials for neonatal BSI episodes.

	***n* (%)**
**Vancomycin**
TDM performed if clinically indicated^a^	39/46 (84.7%)
TDM performed at the correct time^b^	16/39 (41.0%)
**Vancomycin drug concentration^c^**
Toxic	6 (15.4%)
Sub-therapeutic	19 (48.7%)
Appropriate	14 (35.9%)
Dosing adjusted following toxic/sub-therapeutic level	15/25 (60.0%)
Vancomycin stopped^f^ following toxic/sub-therapeutic level	6/25 (24%)
**Amikacin**	
TDM performed if clinically indicated^d^	8/27 (29.6%)
TDM performed at the correct time^e^	2/8 (25.0%)
**Amikacin trough concentration**
<2.5 μg/mL	3 (37.5%)
2.5–5 μg/mL	4 (50.0%)
>5 μg/mL	1 (12.5%)
Amikacin stopped^f^ following trough concentration >2.5 μg/mL	4/5 (80%)
**Gentamicin**	
TDM performed if clinically indicated^d^	1/8 (12.5%)
TDM performed at the correct time^e^	0/1 (0.0%)
**Gentamicin trough concentration**
<1 μg/mL	0 (0.0%)
1–2 μg/mL	0 (0.0%)
>2 μg/mL	1 (100%)
Gentamycin stopped^f^ following trough concentration>2 μg/mL	1/1 (100%)

a *TDM was indicated in any patient where ≥4 doses of vancomycin were administered*.

b *Prior to the 4th dose of vancomycin administration or, if loading dose given, prior to the 3rd or 4th dose of vancomycin administration*.

c *Toxic > 20 μg/mL; sub therapeutic <10 μg/mL; appropriate 10–20 μg/mL*.

d *Any patient where ≥3 doses of amikacin or gentamicin were administered*.

e *Prior to the 3rd dose of amikacin or gentamicin administration*.

f *Drug stopped within 24 h of TDM sample*.

## Discussion

In this cohort of 141 hospitalized VLBW neonates with BSI, we evaluated the prevalence of antimicrobial prescription and administration-related errors for 495 prescriptions. Median hang-time to the first dose of empiric antimicrobial was prolonged beyond 60-min in 64% of prescriptions. Prescription errors (15.6% for dose and 16.4% for interval) were 4-fold more prevalent than administration errors (1.4% had delayed doses and 3.8% had missed doses), particularly in neonates >7 days postnatal age. Prescription errors were frequent for vancomycin (dose errors) and piperacillin-tazobactam (dose interval errors). There was moderate concordance between pathogen susceptibility and empiric antimicrobial choice (75%).

BSI- attributable mortality occurred in almost one-third of neonates (49/141, 30.4%). This is in keeping with a crude mortality of 29% in VLBW infants at Tygerberg hospital in 2015 (5), but much higher than the BSI-attributable neonatal mortality observed in in Taiwan (7%) and China (5–14%) (31, 36). These studies included term and preterm infants, with a predominance of gram positive organisms pathogens. Reasons for the substantially higher BSI-attributable mortality rates at our institution may include: a greater proportion of fulminant gram negative BSI, higher rates of antimicrobial resistance, a greater percentage of preterm neonates with immature immunity (46% of our cohort weighed <1,000 g at birth) and local guidelines that limit access to NICU care for babies with birth weight <800 g (28). A large German study confirmed that gram negative pathogens are associated with higher mortality in VLBW neonates compared to infection with gram-positive organisms (37). Their crude mortality of 5.7% is significantly lower than ours, but only 16% of BSI's were caused by gram-negative organisms in their cohort compared to 73% in our study.

The duration of antimicrobial therapy at our unit was 9 days for primary BSI, but substantially longer in neonates with meningitis and surgical infections. In a systematic review of optimal antibiotic duration in children, 10 days of intravenous treatment is suggested for bacteraemia caused by gram negative organisms and 7–14 days for Staphylococcal infections (38). In many neonatal units there is substantial variability in duration of antimicrobial therapy for infection, and this is a potential target for antimicrobial stewardship to standardize, and where safe to do so, reduce length of therapy (39).

The potential for adverse drug events in neonates is high, especially for patients in the NICU, with 79% occurring at the stage of drug prescribing and 34% involving incorrect dosing in one American study (40). In children, prescribing errors are more commonly found with antimicrobials than other agents (20, 22, 41) possibly due to the fact that, along with sedatives, they are the most common class of drug prescribed (20). This was confirmed locally in a study from Gauteng including NICU patients as well as pediatric inpatients, where 43% of errors were related to anti-infective agents (21). Almost 20% of antimicrobial prescriptions had dose errors with 18.9% frequency errors in a pediatric study in Pakistan (42). The error rates in our VLBW neonatal population are comparable, although slightly lower, at 15.6 and 16.4%, respectively. Our study shows 42% of dose errors were associated with high doses and 57% with low doses. These findings were similar to a French study which reported 46% high dose errors and 54% low dose errors (43).

A Brazilian study noted that in general, prescribing errors are more frequent in preterm neonates with dosing errors occurring in 18.5% of preterm neonates compared to 12.9% in term neonates (18). These rates are still higher than those seen in manual prescriptions for preterm infants, <33 weeks, in France (43) where they subsequently recommended the use of computerized systems for prescribing in neonatal units. Electronic prescriptions in children were studied by Maat et al. and they found that the use of a “standardized structured template” reduced the risk of errors compared to “free text” prescriptions (44). Our unit does not currently have access to electronic prescribing systems, however more simple interventions such as preformatted prescription sheets, training, pharmacist-led prescription reviews and the use of a single reference source have been successful in reducing prescription errors (17, 19, 45, 46). No single intervention to reduce medication errors has been proven to be superior, thus individual units need to implement what is practical and cost-effective in their setting (47).

The only factor that was associated with prescription errors in our cohort was a postnatal age of 7 days or more, where prescriptions for HA-BSI necessitate use of a wider range of antimicrobials, some of which are not commonly used. In addition, some antimicrobial dosing intervals change with increasing postnatal age, necessitating accurate calculation by the prescriber which increases risk of errors. Another factor that may have contributed to confusion among prescribers, is the use of two different neonatal prescribing guidelines, which did not include recommendations for at least three antimicrobials used in our unit. Many doctors work at both tertiary hospitals in the Western Cape and their respective referral hospitals and thus reference books are shared between the units. Development of a single drug dose reference book for all provincial neonatal units should be considered to minimize prescription errors.

Studies in adults and children have focused on the concept of the golden hour for initiation of antimicrobial treatment in patients with sepsis (11). In our study, the median hang-time to empiric antimicrobial was close to 2 h for both early-onset BSI and HA-BSI. In the EOS group, delays in admitting neonates from labor ward or theater may have been a contributing factor. In patients with HAI, difficulty obtaining intravenous access and poor communication between medical and nursing staff may have contributed to prolonged hang-times. In our neonatal wards there are limited number of registered nurses able to administer medication, thus if the sepsis diagnosis is not directly communicated to them, there is an increased chance that they will only come across the medication chart when doing their medication rounds. In a quality improvement study in South Carolina, improved communication successfully reduced antimicrobial hang-time in neonatal HAI (48). In contrast to our findings, a NICU sepsis study found prolonged hang-time was an independent risk factor for death (12). However, their patients were bigger (mean birth weight 2.3 kg, mean GA 34 weeks), and isolated mostly gram positive pathogens. In our setting and in Germany gram negative infections have shown significant association with mortality (5, 37). Possible reasons for the failure to find an association between hang-time and mortality in our cohort may be the small sample size and the predominance of gram negative BSI pathogens with a fulminant disease course, despite prompt administration of antimicrobials.

Cook et al. showed that neonates and children receiving discordant empiric antibiotics had a 3-fold higher risk of death within 30 days of BSI (49) but this was not found in our study. Only 15.5% of their cohort were preterm neonates. Death within 72 h of sepsis diagnosis was not significantly higher in our patients who received discordant empiric therapy. Although not significant, there appeared to be a longer delay in initiating concordant therapy in HA-BSI episodes, likely due to higher rates of antimicrobial resistance. Most antimicrobials are kept as ward stock for rapid access, however in some cases of less commonly used antimicrobials (e.g., linezolid, imipenem), delays may occur while waiting for pharmacy to issue the drugs or whilst awaiting permission from the antimicrobial stewardship committee (e.g., colistin).

In a review of medication incidents in England and Wales, omitted and delayed doses were the most common category of error reported (50). Comparatively the percentage of missed and delayed antimicrobial doses in this study was low at 3.8 and 1.4%, respectively. This may be a spurious finding as antimicrobial administration was not observed in our study. Thus, correlation between doses signed for and the actual time of administration could not be confirmed. In a busy neonatal unit with low nurse-to-patient ratios, one could postulate that antimicrobials with less frequent dosing would have fewer administration errors. This is in keeping with our once daily aminoglycoside dose recommendation.

In this study, although vancomycin TDM was done regularly, it was only done at the recommended time in 41% of cases. This may have been due to limited staff availability after hours to collect blood samples or, to cluster blood sampling to minimize the number of neonatal invasive procedures. Additionally, vancomycin dose adjustments often require regular consultations with clinical pharmacologists whose services are only available in large, academic centers contributing to inadequate dosing/ dosing errors. The aminoglycoside TDM was less well-performed and is an area where management can be improved. Medical and nursing staff training, close review of prescription charts on ward rounds and antimicrobial stewardship rounds, and inclusion of TDM recommendations in antimicrobial guidelines and unit protocols should be implemented.

This study has several limitations including a retrospective design, unavailability of some prescription charts (2.5%) and the lack of documentation of time of BSI diagnosis in the clinical notes (time of blood culture sampling recorded on the laboratory information system was used as a proxy). However, this resulted in some episodes where antimicrobials appeared to have been given before infection diagnosis, and were thus excluded from the hang-time analysis. In addition, we were unable to identify time of prescription (day/night) and the seniority of the prescribing doctor as that information is not currently captured on our antimicrobial prescription charts. One might postulate that junior doctors may be more prone to prescribing errors, or that after-hours, when there is less support, errors may be more frequent. The quality of the drug prescription in terms of legibility, unspecified data etc was not described as a standardized, structured prescription chart is used to reduce these issues. Information regarding whether antimicrobials were administered in the wards vs. the NICU, with potentially better nurse to patient ratios, was also not available. As the study was retrospective, the preparation and administration of drugs was not observed, thus the accuracy of antimicrobial time of administration could not be confirmed, which may account for the low frequency of administration errors. The strength of this study, however, lies in the large number of prescriptions analyzed and the detailed analysis of several prescription related metrics. In addition there is very limited data available for this population (VLBW neonates with BSI from SSA), a group known to be at higher risk of BSI. This study provides novel data and comprehensive analysis to address this gap. The findings of this study could potentially guide other neonatal units facing similar challenges with antimicrobial prescription and administration.

## Conclusion

Although antimicrobial prescription and administration errors are prevalent in this vulnerable population of VLBW neonates, the frequency of errors is similar to that reported from other lower and upper-middle income countries (LMIC). However, the rates are higher than that observed in high income countries. Further studies are needed to identify and measure the impact of future interventions to reduce antimicrobial prescribing and administration errors in resource-limited neonatal units. Potential prescribing interventions could include use of a standardized antimicrobial reference guide, improved communication to decrease hang-time, more detailed prescription charts, pharmacist-led prescription review and antimicrobial stewardship interventions to reduce duration of therapy and minimize dosing errors.

## Data Availability Statement

The raw data supporting the conclusions of this article will be made available by the authors, without undue reservation.

## Ethics Statement

The studies involving human participants were reviewed and approved by Stellenbosch University Health Research Ethics Committee. Written informed consent from the participants' legal guardian/next of kin was not required to participate in this study in accordance with the national legislation and the institutional requirements.

## Author Contributions

The study was conceptualized by SH and AD. All data collection was done by SH. All authors contributed to the data analysis and manuscript preparation and have read and approved the final manuscript.

## Funding

AD was supported by a National Institutes of Health Emerging Global Leader Award (NIH K43 TW010682).

## Conflict of Interest

The authors declare that the research was conducted in the absence of any commercial or financial relationships that could be construed as a potential conflict of interest.

## Publisher's Note

All claims expressed in this article are solely those of the authors and do not necessarily represent those of their affiliated organizations, or those of the publisher, the editors and the reviewers. Any product that may be evaluated in this article, or claim that may be made by its manufacturer, is not guaranteed or endorsed by the publisher.
